# Forced expiration measurements in mouse models of obstructive and restrictive lung diseases

**DOI:** 10.1186/s12931-017-0610-1

**Published:** 2017-06-19

**Authors:** Fien C. Devos, André Maaske, Annette Robichaud, Lore Pollaris, Sven Seys, Carolina Aznar Lopez, Erik Verbeken, Matthias Tenbusch, Rik Lories, Benoit Nemery, Peter HM Hoet, Jeroen AJ Vanoirbeek

**Affiliations:** 1 Center for Environment and Health, KU Leuven, Leuven, Belgium; 2 Molecular and Medical Virology, Ruhr-University, Bochum, Germany; 3 SCIREQ Scientific Respiratory Equipment Inc., Montreal, QC Canada; 4 Clinical Immunology, KU Leuven, Leuven, Belgium; 5 Skeletal Biology and Engineering Research Center, KU Leuven, Leuven, Belgium; 6 Translational Cell and Tissue Research, KU Leuven, Leuven, Belgium; 7 Institute of Clinical and Molecular Virology, University Hospital Erlangen, Erlangen-Nürnberg, Germany

**Keywords:** Fibrosis, Emphysema, Asthma, Acute lung injury, Mice, Forced oscillations technique, Negative pressure forced expiration, Airway resistance, Tissue elastance, Forced expiratory volume, Forced vital capacity

## Abstract

**Background:**

Pulmonary function measurements are important when studying respiratory disease models. Both resistance and compliance have been used to assess lung function in mice. Yet, it is not always clear how these parameters relate to forced expiration (FE)-related parameters, most commonly used in humans. We aimed to characterize FE measurements in four well-established mouse models of lung diseases.

**Method:**

Detailed respiratory mechanics and FE measurements were assessed concurrently in Balb/c mice, using the forced oscillation and negative pressure-driven forced expiration techniques, respectively. Measurements were performed at baseline and following increasing methacholine challenges in control Balb/c mice as well as in four disease models: bleomycin-induced fibrosis, elastase-induced emphysema, LPS-induced acute lung injury and house dust mite-induced asthma.

**Results:**

Respiratory mechanics parameters (airway resistance, tissue damping and tissue elastance) confirmed disease-specific phenotypes either at baseline or following methacholine challenge. Similarly, lung function defects could be detected in each disease model by at least one FE-related parameter (FEV_0.1_, FEF_0.1_, FVC, FEV_0.1_/FVC ratio and PEF) at baseline or during the methacholine provocation assay.

**Conclusions:**

FE-derived outcomes in four mouse disease models behaved similarly to changes found in human spirometry. Routine combined lung function assessments could increase the translational utility of mouse models.

**Electronic supplementary material:**

The online version of this article (doi:10.1186/s12931-017-0610-1) contains supplementary material, which is available to authorized users.

## Background

At the preclinical level in experimental animals, pulmonary function is generally assessed using the forced oscillation technique (FOT). This is an invasive method allowing accurate measurements of physiologically relevant parameters describing the mechanical properties of the respiratory system. The classical parameters of respiratory mechanics, resistance and compliance, relate to the opposition to air moving in and out of the lungs and to the distensibility of the respiratory system, respectively. These measurements are based on the analysis of pressure, volume, and flow signals acquired in response to oscillatory airflow waveforms applied at the subject’s airway opening [1]. Through the use of complex oscillatory signals and advanced mathematical model fitting, this technique is also able to distinguish between airway and tissue mechanics [2].

FOT measurements have already been used in mouse models of respiratory diseases to investigate pathophysiological changes associated with fibrosis [3], emphysema [4], or asthma [5–8]. Yet, from a pre-clinical perspective, the translational value of the mouse as a model to study respiratory diseases has been challenged and it is not always clear how changes in respiratory mechanics parameters relate to the commonly measured forced expiration (FE) parameters in humans [9]. Nowadays, spirometric-like measures can be performed in mice by rapidly exposing the airways to a negative pressure in order to generate a forced expiratory flow signal [10]. The technique is referred to as negative pressure-driven forced expiration (NPFE) and it can be performed concurrently with FOT measurements, in the same animal using a single device, to obtain, in addition to respiratory mechanics’ parameters, endpoints resembling the clinically used ones, such as forced expiratory volume at 0.1 s (FEV_0.1_), forced vital capactity (FVC), “Tiffeneau” index (FEV_0.1_/FVC) or peak expiratory flow (PEF). As is the case for human subjects, both FOT and FE outcomes can also be measured during a bronchoprovocation test to increasing concentrations of methacholine to determine airway hyperresponsiveness (AHR) [9, 10].

Previously, Vanoirbeek *et al.* [11] reported flow-volume (FV) loops and associated FE parameters measured at baseline in mouse models of asthma, fibrosis and emphysema. In that particular study, FE measurements were performed with a system deprived of any aerosol generation or FOT measurement capabilities. Therefore, in order to be able to assess airway responsiveness to aerosolized methacholine, the subjects had to be moved from the FOT device to the FE system during the course of the experiment. In addition to the technical challenges and a loss in precision in the timing of the measurements, the use of that FE system was also associated with the need to manually construct the FV curves, which were generated from a total of 9 data points acquired during the FE maneuver. More recent FE systems now have the ability to automatically generate FV loops and derive FE parameters. In addition, the FV curves are constructed from higher resolution volume and flow signals (containing approximately 360 data points), thus providing a more accurate and specific assessment of the respiratory system under conditions of greater expiratory pressure gradient. The objective of this study was to evaluate FE endpoints (FEV_0.1,_ FVC, PEF, FEV_0.1_/FVC) as potential diagnostic parameters in different mouse models of respiratory diseases. In order to do so, we seamlessly performed FOT and NPFE measurements at baseline and after increasing concentrations of methacholine using a single measurement device that provided an accurate FE assessment. Since the hypothesis was that FE-related changes would generally behave similarly to changes found in human spirometry across a range of respiratory diseases, we included a model of lung fibrosis, emphysema, and acute lung injury, as well as a model of allergic asthma. All four mouse respiratory disease models were compared to a group of naive control animals.

## Methods

### Reagents

Acetyl-β-methylcholine (methacholine, MCh), cyclophosphamide monohydrate, porcine pancreatic elastase (PPE) and lipopolysaccharide (LPS) were supplied from Sigma-Aldrich (Bornem, Belgium). Formaldehyde (36%) was obtained from VWR international (Leuven, Belgium) and was diluted to 4% in distilled water. Bleomycin sulfate (Bleo) was supplied from Sanofi-Aventis (Diegem, Belgium), Isoflurane (Forene) from Abbott (Ottignies, Belgium), Pentobarbital sodium (Nembutal) from Sanofi Santé animale (CEVA, Brussels, Belgium), Xylazine from VMD S.A. (Arendonk, Belgium) and Ketamine from Eurovet Animal Health (Bladel, Netherlands).

### Animals and disease models

Male BALB/c mice, obtained from Harlan (The Netherlands) at 9 weeks old, were divided into 5 different groups: bleomycin (Bleo)-induced lung fibrosis (*n =* 10), porcine pancreatic elastase (PPE)-induced emphysema (*n =* 10), lipopolysaccharide (LPS)-induced acute lung injury (ALI) (*n =* 8), house dust mite (HDM)-induced allergic asthma (*n =* 7), and an untreated control group (*n =* 8). Due to expected mortality in the Bleo fibrosis and the PPE emphysema groups, we included two extra mice in each one. All mice were housed in a conventional animal facility with 12-h dark/light cycles. They were housed in filter top cages and received lightly acidified water and pelleted food *ad libitum*. All experimental procedures performed in mice were approved by the KU Leuven local Ethical Committee for animal experiments (P166-2012).

#### Fibrosis

To induce pulmonary fibrosis, mice (10 weeks old) received an intraperitoneal injection of cyclophosphamide (100 mg/kg). Two days later, the animals were anesthetized with an intraperitoneal injection of ketamine/xylazine (100 mg/kg ketamine and 10 mg/kg xylazine; 100 μL/10 g) and intratracheally instilled with bleomycin. Bleomycin was dissolved in sterile PBS and administered as a single dose of 0.1 unit/40 μL per animal [11–13]. At the age of 14 weeks, i.e. three weeks after the first bleomycin administration, mice were evaluated (see Additional file 1: Figure S1).

#### Emphysema

Emphysema was induced in mice (9 weeks old) by administration of porcine pancreatic elastase (PPE). PPE was dissolved in sterile saline and administered in a dose of 1.5 units/50 μL per animal by intranasal instillation, after light anesthesia with isoflurane. Mice were subsequently exposed on a weekly basis to 1.5 units PPE via the same administration route during a period of three weeks [11, 12, 14]. At the age of 14 weeks, i.e. three weeks after the last PPE instillation, mice were evaluated (see Additional file 1: Figure S1).

#### Acute lung injury

To induce acute lung injury, mice (13 weeks old), received a single intranasal instillation of LPS. LPS was dissolved in sterile saline and administered as a single dose of 3 μg/40 μL/mouse after light anesthesia [15]. The mice were studied at the age of 13 weeks, i.e. two days after the instillation (see Additional file 1: Figure S1).

#### Allergic asthma

BALB/c mice (11 weeks old) were endonasally sensitized with 1 μg of HDM extract (Greer Laboratories, Lenoir, NC) in 50 μL of saline at day 1. At days 8 to 13, mice were endonasally challenged with 10 μg of HDM extract in 50 μL of saline [16]. Two days after the last challenge, i.e. at the age of 13 weeks, lung function measurements were performed (see Additional file 1: Figure S1).

### Lung function measurements

FOT and FE measurements were performed using the flexiVent FX system (SCIREQ Inc., Montreal Qc, Canada). The system was equipped with a FX1 module as well as with a NPFE extension for mice and it was operated by the flexiWare v7.2 software. A small particle size Aeroneb Lab nebulizer (2.5–4 μm; Aerogen, Galway, Ireland) was integrated in the inspiratory arm of the Y-tubing for the generation of the aerosol challenges. The nebulizer activation was synchronized with inspiration and set to a 50% duty cycle for 5 s. On the day of the experiment, mice were anesthetized with an IP injection of pentobarbital sodium (70 mg/kg body weight). Once a surgical plane of anaesthesia was reached, the trachea was exposed to insert an 18-gauge metal cannula having a typical resistance of 0.3 cmH_2_O.s/mL. Mice were quasi-sinusoidally ventilated with a tidal volume of 10 mL/kg, a frequency of 150 breaths/min, an inspiratory to expiratory ratio of 2:3, and a positive end-expiratory pressure of 3 cmH_2_O.

#### A. Baseline measurements

At the start of an experiment, two deep inflations were successively applied to maximally inflate the lungs to a pressure of 30 cmH_2_O in order to open-up closed areas and standardize lung volume. The lungs were allowed to equilibrate at that pressure over a period of 3 s and the gas compression-corrected volume was read as the subject’s inspiratory capacity (IC), typically on the second maneuver. A broadband forced oscillation waveform inducing frequencies between 0.5 to 19.75 Hz (Prime-8; P8) was then applied to the subject’s airway opening during 8 s. This FOT measurement was analyzed by fitting the constant-phase model to the respiratory input impedance calculated from the P8 experimental data [2]. This mathematical model allows the separate assessment of the airway and tissue contributions to the respiratory response. The Newtonian resistance (R_n_, airway resistance, which is dominated by the resistance of the large conducting airways), tissue damping (G, closely associated to tissue resistance and the resistance of the small peripheral airways), tissue elastance (H), and tissue hysteresivity (eta) (G/H) were considered in this study, provided that the coefficient of determination of the model fit was ≥ 0.9. This was followed by the construction of a pressure-volume (PV) curve using a ramp-style pressure-driven maneuver (PVr-P), from which the static compliance (Cst) was calculated directly from the deflating arm of the PV loop between the pressures of 3 –7 cmH_2_O. Considering the high distensibility of emphysematous lungs, PV loops of mice with emphysema were only reported if the maximum inflation pressure of +30 cmH_2_O was reached. The hysteresis (Area), or area between inflation and deflation limb of the PV curve, was also calculated. Finally, a NPFE maneuver was performed to obtain a FV loop and the FE-related parameters. This was done by first inflating the lungs to a pressure of +30 cmH_2_O over 1.2 s and then rapidly exposing the subject’s airways to a negative pressure of -55 cmH_2_O to generate an imposed negative expiratory pressure gradient. From the FE parameters, the forced expiratory volume and flow at 0.1 s (FEV_0.1_, FEF_0.1_, respectively), forced vital capacity (FVC), and peak expiratory flows (PEF) were considered in the present study as well as the FEV_0.1_/FVC (referred to as Tiffeneau index in humans at 1 s).

#### B. Airway responsiveness assessment

After performing the sequence of measurements at baseline level, a protocol for measuring airway responsiveness (AHR) to methacholine (MCh) was initiated. Each mouse was exposed for 5 s to an aerosol of a solution of MCh in increasing concentrations (0, 1.25, 2.5, 5, 10, 20 mg/mL). Starting almost immediately after the end of the aerosol exposure, an automated sequence of closely-spaced FOT measurements was initiated using the 3 s long, broadband forced oscillation perturbation ‘Quick Prime-3’ (QP3). Five consecutive QP3 perturbations were run at approximately 15 s apart, resulting in 5 measurements for each concentration of MCh. Each sequence was followed by a NPFE measurement taken approximately 15 s after the last FOT measurement. The dose-response curve to MCh was constructed in a cumulative manner with no recruitment maneuvers in between aerosol challenges as this approach had the advantage of enabling the separation between control and disease mice. Since the NPFE-induced derecruitment of lung units was not reversed by a deep lung inflation maneuver in between aerosol challenges and that it was shown to specifically affect the tissue-related parameters G and H [10], R_n_ was the only FOT-related parameter considered in assessing airway responsiveness to MCh in the present study to avoid any confounding effects.

For each mouse of the LPS-ALI, HDM-asthma and control group, the provocative concentration of MCh inducing either a 20 (PC20), 30 (PC30) or 40% (PC40) decrease in FEV_0.1_ was assessed, by calculating the slope of the dose-response curve of each individual mouse, where the peak responses to MCh were normalized to the FEV_0.1_ of 0 mg/ml MCh (=100%).

### Inflammatory cells in the bronchoalveolar lavage fluid

Immediately after the lung function measurements, the lungs were lavaged, *in situ*, three times with 0.7 mL sterile saline (0.9% NaCl), and the recovered fluid was pooled. Cells were counted using a Bürker hemocytometer (total cells) and the bronchoalveolar lavage (BAL) fluid was centrifuged (1000 g, 10 min). For differential cell counts, 250 μL of the resuspended cells (100,000 cells/mL) were spun (1400 g, 6 min) (Cytospin 3, Shandon, TechGen, Zellik, Belgium) onto microscope slides, air dried and stained (Diff-Quik® method, Medical Diagnostics, Düdingen, Germany). For each sample, 200 cells were counted for the number of macrophages, eosinophils, neutrophils and lymphocytes.

### Histopathology

The lungs were fixated in 4% of formaldehyde. After dehydration and embedding in paraffin, sagittal sections were stained with hematoxylin and eosin (H&E).

### Statistical analyses

For each mouse, the average of all measurements was calculated for R_n_, grouped per experimental conditions, and plotted against MCh concentration. The results are presented as mean ± standard deviation. MCh dose-response curves (R_n_, FEV_0.1_, and FVC) were analyzed using a two-way parametric analysis of variance (ANOVA) followed by a Bonferroni multiple comparison *post hoc* test to compare each treatment group with the control group. The area under the MCh dose-response curve (AUC) was also calculated and statistically analyzed using a one-way parametric ANOVA. All other statistical analyses were performed using unpaired t-tests, to compare each disease entity separately with the control group (GraphPad prism 5.01, Graphpad Software Inc., San Diego, CA).

## Results

During the induction protocols, two mice of the PPE-treated (emphysema) group and two mouse of the Bleo-treated (fibrosis) group died. In both groups, mice had significantly lower body weights on the day of analysis, compared with the control group. In the HDM asthma group, one mouse was excluded because all data were far out of the 95th percentile of the group. All data reflect measurements from at least seven mice per group.

### Inflammatory cells in BAL fluid

**Fig. 1:**
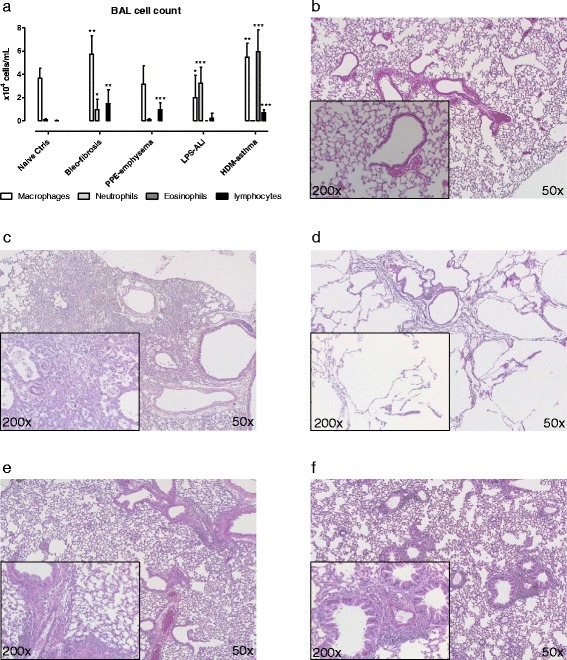
Differential cell counts in bronchoalveolar lavage and histological analyses. Total numbers of macrophages, neutrophils, eosinophils and lymphocytes were determined in the bronchoalveolar lavage (BAL) fluid, obtained immediately after lung function measurements (**a**). Next, lungs were isolated and slides of the lungs from mice with different lung diseases were prepared and colored with H&E-staining for analysis at a magnification of 50× and 200×, shown for naive control group (**b**), Bleo-fibrosis (**c**), PPE-emphysema (**d**), LPS-ALI (**e**) and HDM-asthma (**f**). Data are presented as mean ± SD. * *p <* 0.05, ** *p <* 0.01 and *** *p <* 0.001 compared to the naive control group. *n =* 7 –8 per group

Compared to the control group, significantly higher numbers of neutrophils were found in the BAL-fluid of mice with bleomycin-induced fibrosis and LPS-induced acute lung injury (ALI). Similarly, significantly higher numbers of eosinophils were found in mice with HDM-induced asthma, in comparison to naive control mice. Lymphocytes were significantly increased in mice with bleomycin-induced fibrosis, elastase-induced emphysema and HDM-induced asthma, compared to naive controls (Fig. 1a).

### Histopathology

To further confirm the underlying pathology, paraffin sections of the lungs were obtained of every mouse. Figure 1 shows an illustrative example of each group. Compared to naive control mice (Fig. 1b), mice with fibrosis presented with a heterogeneous inflammatory cell infiltrate in both the broncho-vascular bundle and alveolar parenchyma, and *fibrous* thickening of *alveolar* septa (Fig. 1c), whereas mice with emphysema presented with a typical heterogeneous destruction of the pulmonary parenchyma (Fig. 1d). Mice with ALI showed a mild infiltration of inflammatory cells, including mononuclear cells and granulocytes in the broncho-vascular bundle and intra-alveolar (Fig. 1e). Mice with asthma showed a pronounced infiltration of mononuclear cells and granulocytes in the broncho-alveolar bundle and in the venous vascular tree. Mild intra-alveolar inflammation was also present (Fig. 1f).

### Lung function measurements

#### A. Baseline measurements

A complete overview of all parameters measured at baseline is reported in the Additional file 2.

##### FOT respiratory mechanics measurements

**Fig. 2:**
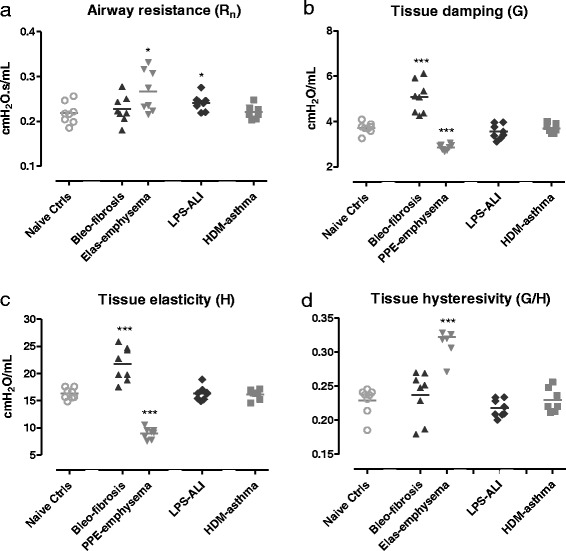
FOT respiratory mechanics parameters at baseline. Central airway resistance, R_n_ (**a**), tissue damping, G (**b**), tissue elastance, H (**c**) and tissue hysteresivity G/H (eta) (**d**) were measured using a prime-8 perturbation. Parameters are shown for each individual mouse, along with group means. * *p <* 0.05 and *** *p <* 0.001 compared to the naive control group. *n =* 7 –8 per group

Consistent with an anticipated increase in tissue stiffness and elastic recoil, mice treated with bleomycin displayed, at baseline, elevated values of G (tissue damping) and H (tissue elastance) relative to naive control mice, with no statistical difference in R_n_ (central airway resistance) (Fig. 2). In contrast, in the PPE-treated mice, where a decrease in elastic recoil was expected, G and H were significantly decreased compared to control animals, resulting in a significantly increased tissue hysteresivity (G/H). These animals also showed a significant increase in airway resistance (R_n_) in comparison to the control group (Fig. 2a). As seen in Fig. 1, mice with ALI also presented with an increase in R_n_, compared to the naive healthy mice. Baseline parameters of mice with HDM-induced asthma did not differ from those of naive control mice (Fig. 2).

##### Pressure-volume loop and inspiratory capacity

**Fig. 3:**
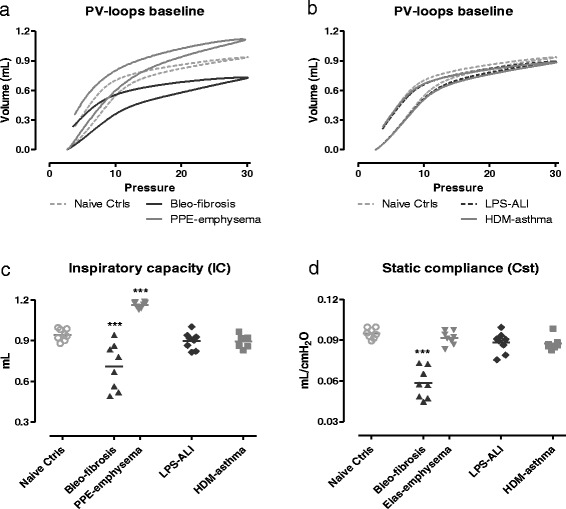
Pressure-volume curves and related parameters at baseline. Pressure-volume (PV) loops were generated using a ramp-style pressure-volume maneuver (PVr-P). Mean PV loops are shown for each group, Bleo-fibrosis and PPE-emphysema in (**a**), LPS-ALI and HDM-asthma in (**b**), and represented together with the naive control group. Inspiratory capacity (**c**) was measured using a deep inflation maneuver, and the static compliance (**d**) was calculated directly from the deflating arm of the PV loop between 3 –7 cmH_2_O. Both parameters are shown for each individual mouse, along with group means. *** *p <* 0.001 compared to the naive control group. *n =* 7 –8 per group

The PV loop of mice with fibrosis showed a characteristic downward shift, while those of emphysematous mice displayed a typical upward shift, compared to naive control mice (Fig. 3a). Consistent with an increased stiffness of the lungs, mice with fibrosis showed a significant decrease in IC and Cst compared to naive controls (Fig. 3c, d). Emphysematous mice, on the other hand, showed a statistically elevated IC with, however, no significant change in Cst relative to the control mice (Fig. 3c). The PV loops from the mice with ALI or with asthma appeared very similar to those from the naive control ones (Fig. 3b). Consistently, IC and Cst were unchanged across these experimental groups (Fig. 3c and d).

##### FE measurements

**Fig. 4:**
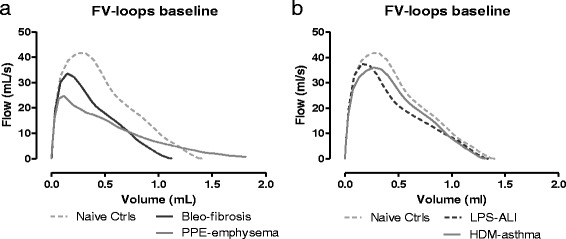
Flow-volume loops at baseline. Negative pressure-driven forced expiration maneuvers were performed at baseline to generate flow-volume (FV) loops. Mean FV loops are shown for each group. Bleo-fibrosis and PPE-emphysema, both parenchymal disorders, are shown in (**a**), LPS-ALI and HDM-asthma are represented in (**b**), together with the naive control group. *n =* 7 –8 per group

Forced expiration in mice with fibrosis resulted in a characteristic FV loop compared to control mice, with reduced PEF and FVC suggestive of restrictive lung disease (Fig. 4a). FV curves of emphysematous mice also displayed a pronounced decrease in PEF with, however, an increased FVC relative to control healthy mice (Fig. 4a). The FV curves of mice with ALI and asthma generally followed the curve of control mice, with some reductions in PEF (Fig. 4b).

**Fig. 5:**
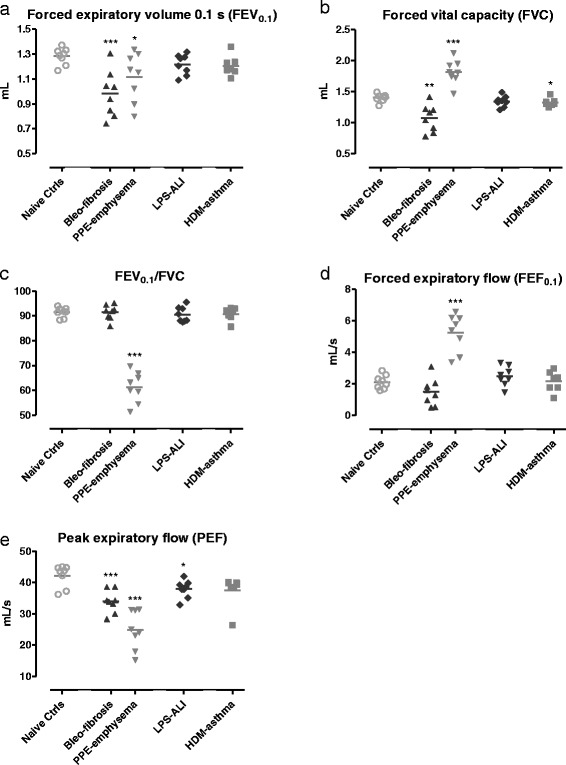
FE-derived parameters at baseline. Forced expiratory volume at 0.1 s, FEV_0.1_ (**a**), forced vital capacity, FVC (**b**), FEV_0.1_/FVC ratio (**c**), forced expiratory flow at 0.1 s, FEF_0.1_ (**d**), and peak expiratory flow, PEF (**e**) were measured in mice by negative pressure-driven forced expiration. Parameters are shown for each individual mouse, along with group means. * *p <* 0.05, ** *p <* 0.01 and *** *p <* 0.001 compared to the naive control group. *n =* 7 –8 per group

These changes in the shape of the FV loops were also reflected in the FE-derived parameters (Fig. 5). Consistent with a typical restrictive airway pattern seen in human patients, mice with fibrosis showed at baseline a significantly reduced FEV_0.1,_ FVC and PEF, as well as a normal FEV_0.1_/FVC ratio (Fig. 5). In mice with emphysema FVC and FEF_0.1_ were significantly increased, while FEV_0.1_, PEF and FEV_0.1_/FVC ratio were significantly decreased. From all FE-related parameters, PEF was significantly decreased in mice with ALI, compared to naive controls. HDM-induced asthma mice displayed at baseline FE-derived parameters that generally followed what was seen in the control mice, with the exception of a small but statistically significant increase in FVC.

#### B. Assessment of airway responsiveness to methacholine

In this B section, we report MCh responses exclusively for mice with LPS-induced ALI and HDM-induced asthma, as these provide clinically-relevant information.

##### FOT respiratory mechanics measurements

**Fig. 6:**
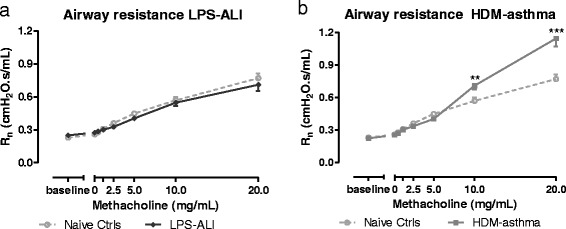
Airway responsiveness to methacholine. Airway resistance (R_n_) was measured in response to increasing aerosol challenges of methacholine (0 –20 mg/mL). Mean (± SD) concentration-response curves of R_n_ are shown for LPS-ALI in (**a**) and HDM-asthma in (**b**), and represented together with that of the naive control group. ** *p <* 0.01 and *** *p <* 0.001 compared to the naive control group.*n =* 7 –8 per group

AHR, expressed in terms of central airway resistance (R_n_), was absent in mice with ALI (Fig. 6a), but present, as expected, in mice with asthma (Fig. 6b). In this latter group, R_n_ was significantly increased at the two highest concentrations of MCh tested, compared to naive control mice (Fig. 6b).

##### FE measurements

**Fig. 7:**
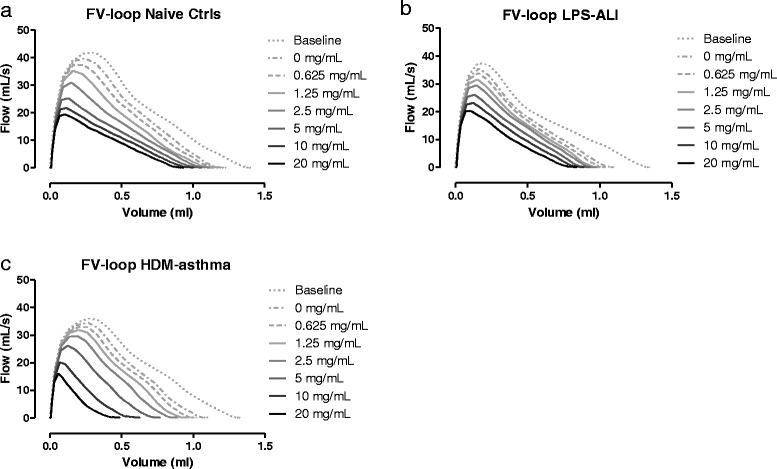
Flow-volume loops. Negative pressure-driven forced expiration maneuvers were performed at baseline and following each methacholine aerosol challenge (0-20 mg/mL) to generate 8 successive FV-loops for each group. Mean FV-loops are shown for Naive Ctrls in (**a**), LPS-ALI in (**b**) and HDM-asthma in (**c**).*n =* 7 –8 per group

Mice with ALI displayed a similar FV loop shape profile as the control ones in response to increasing MCh aerosol challenges (Fig. 7a,b). The shape of the FV loops of the mice with HDM-induced asthma became more concave with increasing concentrations of MCh, reflecting a typical pattern of airflow limitation due to obstruction. The FV curve of the HDM-treated group also showed a decreasing trend in PEF and in FVC relative to control mice with increasing concentrations of MCh (Fig. 7a,c).

**Fig. 8:**
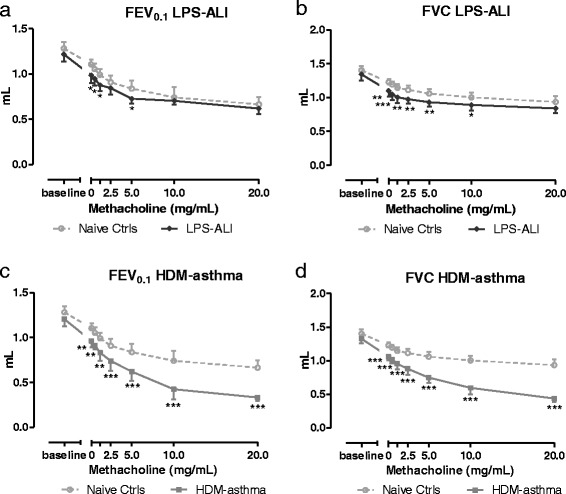
FE-derived parameters. Negative pressure-driven forced expiration maneuvers were performed at baseline and following each methacholine aerosol challenge (0 –20 mg/mL). Forced expiratory volume at 0.1 s, FEV_0.1_ (**a**, **c**), forced vital capacity, FVC (**b**, **d**) are shown for LPS-ALI (**a**, **b**) and HDM-asthma (**c**, **d**). The mean (± SD) concentration-response curve of each group is represented together with that of the naive control group. * *p <* 0.05, ** *p <* 0.01 and *** *p <* 0.001 compared to naive controls. *n =* 7 –8 per group

As for the associated NPFE-related parameters, all groups showed a decreasing trend in FEV_0.1_ and/or FVC with increasing concentrations of MCh (Fig. 8). In mice with ALI, FEV_0.1_ was significantly reduced at 0, 0.6, 1.25 and 5 mg/mL of MCh, compared to control mice (Fig. 8a) although the differences between the two groups were small. Also, FVC was significantly reduced over a similar range of MCh concentrations (Fig. 8b). The FEV_0.1_/FVC ratio of mice with ALI did not differ from naive controls (see Additional file 3: Figure S2). In mice with HDM-induced asthma, both FEV_0.1_ and FVC were significantly lower at all MCh concentrations compared to control mice (Fig. 8). The curves of FEV_0.1_/FVC ratio of mice treated with HDM, followed the curve of control mice (see Additional file 3: Figure S2).

**Fig. 9:**
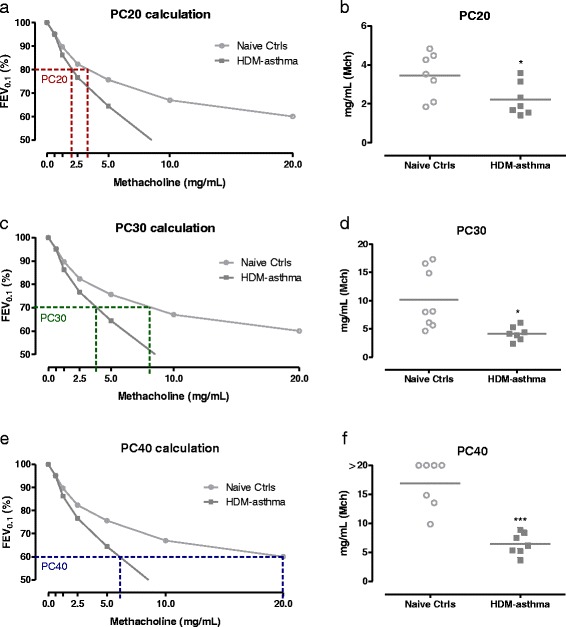
Provocative methacholine concentrations in mice. Mean FEV_0.1_ concentration-response curves to MCh are shown for HDM-asthma and naive controls, with indication of corresponding provocative concentration inducing a 20% (PC20) (**a**), 30% (PC30) (**c**), or 40% (PC40) (**e**) decrease in baseline FEV_0.1_. PC20, PC30, and PC40, calculated from the concentration-response data for each individual mouse and group means are shown in **b**, **d**, and **f**, respectively. * *p <* 0.05, *** *p <* 0.001 compared to naive control group. *n =* 7 –8 per group

The provocative concentration of MCh required to induce a 20, 30 or 40% decrease in baseline FEV_0.1_, was significantly lower in mice with HDM-induced asthma compared to naive controls (Fig. 9). In mice with ALI, no changes in provocative concentrations of MCh were found, compared to naive controls (data not shown).

## Discussion

The lung carries many functions within the body, the most important being that of providing a constant supply of O_2_ / removal of CO_2_ through the process of respiration. This function of the lung is essentially a mechanical one, as it relies on changes in pressure and volume. While it is highly influenced by the mechanical properties of the respiratory system, the lung can very often continue to carry it out under tidal volume conditions despite significant histologic disease manifestations. Therefore, it is frequently necessary to study the lung under different conditions in order to detect pathological lung function defects. In the present study, we performed FE measurements in four well-established mouse models of respiratory diseases with the objective of further assessing disease-specific changes using FE-derived parameters. Studying the lung under forced expiration assesses its function under conditions susceptible of inducing expiratory flow limitation. Combined with measurements of respiratory mechanics, specific lung volumes, and quasi-static pressure-volume curves, FE measurements can potentially add valuable information and contribute to produce a more comprehensive functional assessment. This approach, which explores the linear as well as the non-linear behaviors of the lung, might be particularly important in mouse models, given the relatively large airways that mice have for their body size [17, 18].

In the present study, all these measurements were performed at baseline as well as in response to MCh provocations, in order to study the lungs under a greater level of functional impairment. The measurement device used offered a refined study design in the sense that all measurement types could be made seamlessly in the same subject, with no need to move it during the course of the experimental protocol or other inconveniences associated to the use of separate pieces of equipment [11]. In addition, the raw signals generated during the forced expiration maneuver were of improved quality relative to previously published work [11].

FOT measurements at baseline proved to be useful to assess lung function in models of pulmonary fibrosis [13] and pulmonary emphysema [12, 14] and confirm the expected changes in respiratory mechanics. The restrictive pathology, observed in mice with fibrosis, was also reflected in FE-related parameters. FEV_0.1_, FVC and PEF were significantly decreased in mice with fibrosis, and could, thus, serve as diagnostic parameters having some similarities with those routinely used in the clinic. This is in contrast to the earlier study of Vanoirbeek *et al.*, where less changes in FE-related variables could be observed in mice with fibrosis [11]. In line with the current study, Tanaka *et al.* reported an increase in respiratory elastance and tissue elastance, as well as a decrease in FVC in mice with bleomycin-induced pulmonary fibrosis [19].

Characterization of the pathology of pulmonary emphysema in mice treated with PPE showed the expected decrease in G and H, and an upward shift in the PV loop, reflecting decreased elastic recoil. Consistently, there was an increase in the inspiratory capacity and a decrease in PEF, but no significant change in Cst in our model, compared to naive healthy mice. Furthermore, in contrast to the human disease situation, FVC was increased, resulting in a decreased FEV_0.1_/FVC ratio [20, 21]. These results correspond with those of Tanaka *et al.*, who also assessed lung mechanics and respiratory dysfuntion in mice with PPE-induced pulmonary emphysema, in terms of tissue elastance and FEV_0.05_, using FOT and NPFE techniques, respectively [22]. Similar to our results, they detected a pronounced decrease in both tissue elastance and FEV_0.05_ under baseline conditions. The lack of change in Cst observed in our study could be related to the model used, the style of the PV loop used (ramp versus plateau style), or alternatively, to the fact that the PV loop was constructed over the inspiratory capacity range instead of over the full lung volume range [23].

The FE-derived parameters discussed in this study, although similar to those reported in humans, were generated by negative pressure. This methodology differs from the technique used with patients and could possibly be responsible for some of the discrepancies observed. In the PPE-treated mice, FEF_0.1_ was significantly increased compared to control mice and therefore differs from what is typically observed in human subjects [20, 21]. Airway collapse and expiratory flow limitation are important factors influencing forced expired flow and these are sensitive to changes in the mechanical properties of the respiratory system. As shown by a significant decrease in H, PPE-treated mice displayed an increased lung compliance which, under negative pressure-driven forced expiration, most likely led to the creation of a choke point at a higher location in the airway tree than in the control mice, thus resulting in a higher upstream airway calibre and therefore a higher expiratory flow at the selected time and negative pressure.

We also included a model of acute lung injury (ALI), induced by endotoxin (LPS) exposure and characterized by acute neutrophilic inflammation. FEV_0.1_ remained unchanged in this mouse model. However, a significant increase in R_n_ was observed in the LPS-ALI mice compared to control ones, as well as a significant decrease in PEF, demonstrating the value of these measurements.

As shown before [11], mouse models of airway inflammation and airway hyperresponsiveness often display normal values for respiratory mechanics parameters measured under baseline conditions. Similarly, R_n_ and H were not affected in mice with HDM-induced asthma. Also in parallel to the study of Vanoirbeek *et al*. [11], Cst and IC did not differ from naive controls under baseline conditions.

As expected, enhanced MCh airway responsiveness relative to control mice could be demonstrated in mice treated with HDM (i.e. increased central airway resistance, R_n_). In mice with ALI, the absence of MCh hyperresponsiveness in terms of R_n_ is in line with the findings of Secher *et al.* [24] in male C57BL/6 mice. However, this latter study contradicts the work of Card *et al.* [25], in which it was reported that LPS-treated male C57BL/6 mice, and not female, exhibit an exaggerated MCh-evoked airway response. There are some small technical differences that distinguish these studies, however, the lack of AHR in the present model of neutrophilic inflammation further confirms the existence of a dissociation between recruitment of inflammatory cells and the development of AHR [26–28].

In this study, we also measured airway responsiveness to MCh in terms of FE-derived variables. In contrast to the study of Shalaby *et al.* [10], we did not perform a deep inflation after each FE measurement to reverse the derecruitment of lung units caused by the NPFE maneuver in order to be able to measure airway responsiveness in the HDM-asthma model over a low methacholine concentration range. Mucus is often present in the airways of allergically inflamed mice and methacholine is a known mucus secretagogue [29]. That technical difference most likely enabled us to reach a greater level of airway functional impairment at any given challenges by limiting the subsequent amount of aerosol delivered to the peripheral airways and thus favoring a more proximal deposition. Despite of these differences in protocols, the shape of the FV loops generated in the present study appeared generally similar to those previously reported, with a typical MCh-evoked cumulative decrease in FEV_0.1_ and FVC. The FV loops decreased gradually, with a more concave shape with increasing MCh concentrations, reflecting an increasing degree of airflow obstruction. The assessment of airway responsiveness using the clinically-resembling parameters FEV_0.1_ or FVC revealed functional changes in the HDM-model of asthma studied, as well as in the LPS-ALI one. Although in that latter model, the separation between groups was much smaller compared to the HDM-model, as also seen with the FOT technique. The difference in the level of statistical significance observed between the two measurement techniques in the LPS-ALI model could be related to the fact that small group sizes were analyzed or, alternatively, to the possibility that FOT and FE measurements are influenced by different determinants or determinant distributions [10]. In agreement with the latter point, there was no statistical difference between the LPS-ALI and control groups when FE results were normalized to the 0 mg/ml (saline) challenge (data not shown). The FEV_0.1_ response curves also allowed, at least in the HDM-induced asthma model, the calculation of the provocative MCh concentration inducing a 20 (PC20), 30 (PC30), or 40% (PC40) decrease in FEV and a classification of the subjects’ AHR response as either mild, moderate, or severe, as performed clinically with human data [30, 31].

The addition of FE measurements to other types of lung function evaluations (e.g. FOT, PV loops, specific volumes) contributed, in the present study, to produce a more complete functional characterization of four well established respiratory disease models, all generated on the same genetic background. This approach helped confirm disease specific phenotypes while further assessing distinctive changes using a number of clinically-resembling parameters. The translational utility of these latter parameters to human spirometry still remains to be established and their interpretation is also not fully understood. However, non-linear behaviors of the lung, such as expiratory flow limitation, often play important roles in respiratory diseases and their evaluation should be, in our view, more frequently included in the evaluation of preclinical models as well as extended. For example, future studies could consider, more chronic models, different genetic backgrounds, as well as additional lung function measurements (e.g. additional lung volumes) or conditions under which measurements are made (e.g. different PEEP or negative pressure).

## Conclusion

In conclusion, four well established mouse models of respiratory diseases were characterized with the objective of further assessing disease-specific changes using FE-related parameters. The results showed that lung function defects could be detected in each disease model by at least one FE-derived parameter under baseline or bronchoconstricted conditions and that the changes observed generally behaved in a similar manner as in human patients. Routinely exploring the linear and non-linear lung behaviors in order to produce more comprehensive and insightful lung function assessments in mouse models could be key in increasing the translational utilities of preclinical studies.

## Additional files

Additional file 1: Figure S1. Experimental design of different airway disease models. Mice were sacrificed at the age of 13-14 weeks (indicated in red). (PPTX 111 kb)

Additional file 2: Table S1. Complete overview of all lung function parameters, measured at baseline using the flexivent FX. (DOCX 23 kb)

Additional file 3: Figure S2. Forced expiration-derived parameters. Negative pressure-driven forced expiration maneuvers were performed at baseline and following each methacholine aerosol challenge (0 –20 mg/mL). FEV0.1/FVC ratio is shown for LPS-ALI (a) and HDM-asthma (b). The mean (± SD) concentration-response curve of each group is represented together with the naive control group. *n =* 7 –8 per group. (PPTX 131 kb)
