# Supplementary material for: Forced expiration measurements in mouse models of obstructive and restrictive lung diseases
Source: Respir Res. 2017 Jun 19;18:123. doi: 10.1186/s12931-017-0610-1 (PMC5477381; doi:10.1186/s12931-017-0610-1)
Supplement: Supplementary file 1 — Experimental design of different airway disease models. Mice were sacrificed at the age of 13-14 weeks (indicated in red). (PPTX 111 kb) [file 12931_2017_610_MOESM1_ESM.pptx]

## Slide 1
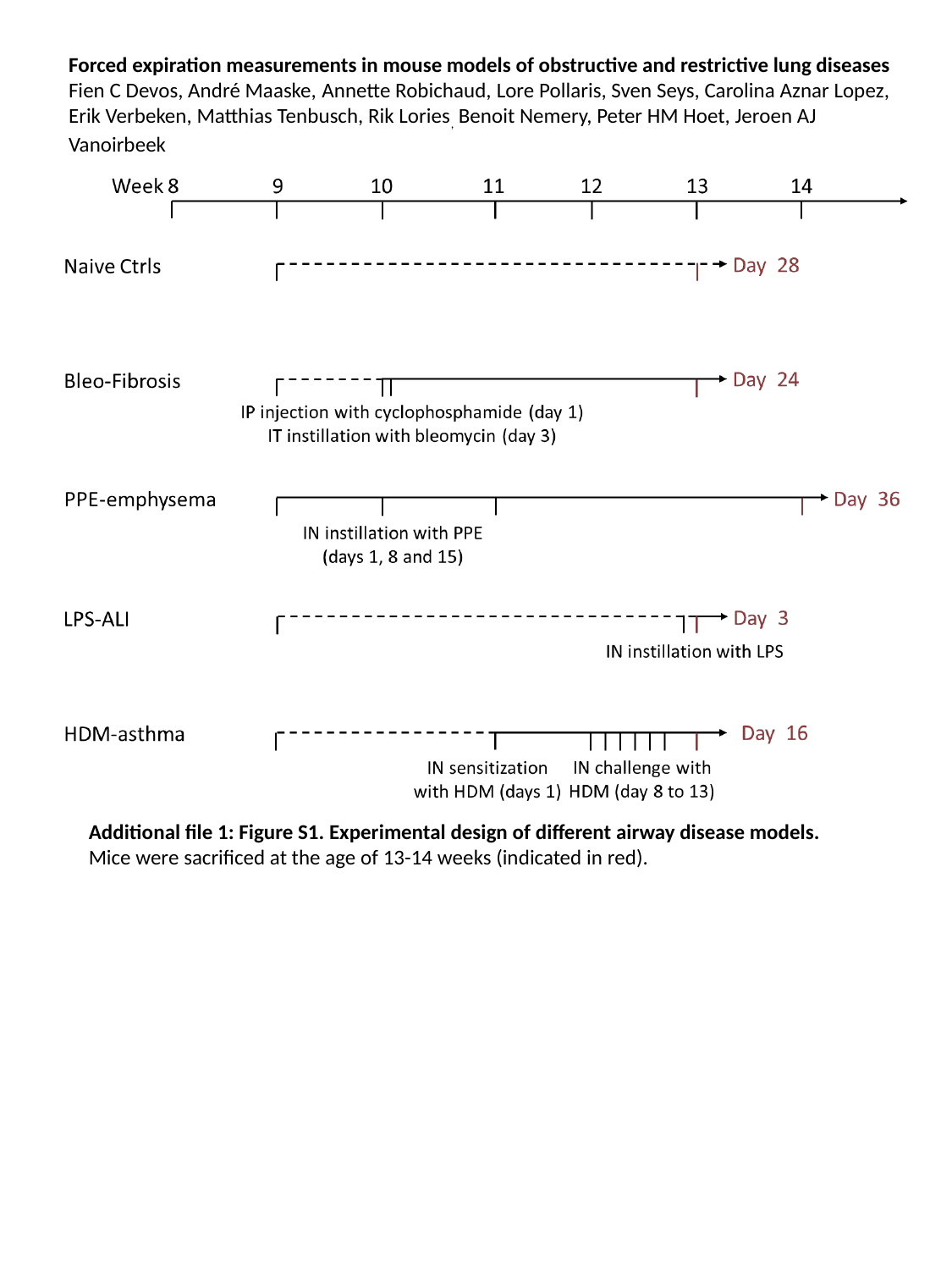

Forced expiration measurements in mouse models of obstructive and restrictive lung diseases
Fien C Devos, André Maaske, Annette Robichaud, Lore Pollaris, Sven Seys, Carolina Aznar Lopez, Erik Verbeken, Matthias Tenbusch, Rik Lories, Benoit Nemery, Peter HM Hoet, Jeroen AJ Vanoirbeek
Additional file 1: Figure S1. Experimental design of different airway disease models.
Mice were sacrificed at the age of 13-14 weeks (indicated in red).
