# Supplementary material for: Forced expiration measurements in mouse models of obstructive and restrictive lung diseases
Source: Respir Res. 2017 Jun 19;18:123. doi: 10.1186/s12931-017-0610-1 (PMC5477381; doi:10.1186/s12931-017-0610-1)
Supplement: Supplementary file 2 — Complete overview of all lung function parameters, measured at baseline using the flexivent FX. (DOCX 23 kb) [file 12931_2017_610_MOESM2_ESM.docx]

**Additional 2: Table S1:** Complete overview of all lung function parameters, measured at baseline using the flexivent FX

| **Task** | **Parameter** | **Unit** | **Experimental groups** | | | | |
| --- | --- | --- | --- | --- | --- | --- | --- |
|  |  |  | **Naive controls** | **Bleo-fibrosis** | **PPE-emphysema** | **LPS-ALI** | **HDM-asthma** |
|  |  |  | Mean ± SD  (n = 8) | Mean ± SD  (n = 8) | Mean ± SD  (n = 8) | Mean ± SD  (n = 8) | Mean ± SD  (n = 7) |
|  | Body weight | g | 27.05 ± 1.05 | 23.06 ± 2.86*** | 24.75 ± 1.76* | 26.36 ± 0.84 | 27.06 ± 1.58 |
| *Deep inflation* | IC | mL | 0.94 ± 0.04 | 0.71 ± 0.17 ** | 1.17 ± 0.02 *** | 0.9 ± 0.06 | 0.9 ± 0.05 |
| *Prime-8* | R_n_ | cmH_2_O.s/mL | 0.22 ± 0.02 | 0.23 ± 0.03 | 0.27 ± 0.05 * | 0.24 ± 0.02 * | 0.22 ± 0.02 |
|  | G | cmH_2_O/mL | 3.72 ± 0.24 | 5.09 ± 0.71 *** | 2.86 ± 0.13 *** | 3.56 ± 0.32 | 3.70 ± 0.21 |
|  | H | cmH_2_O/mL | 16.31 ± 1.02 | 21.74 ± 3.09 *** | 8.97 ± 1.01 *** | 16.34 ± 1.26 | 16.16 ± 0.94 |
|  | G/H (eta) |  | 0.23 ± 0.02 | 0.24 ± 0.04 | 0.32 ± 0.03 *** | 0.22 ± 0.01 | 0.23 ± 0.02 |
| *PVr-P* | Cst | mL/cmH_2_O | 0.10 ± 0.004 | 0.06 ± 0.01 *** | 0.09 ± 0.005 | 0.09 ± 0.008 | 0.09 ± 0.005 |
|  | Area | cmH_2_O.mL | 2.34 ± 0.18 | 3.62 ± 2.42 | 3.97 ± 0.60 *** | 2.18 ± 0.86 | 2.45 ± 0.30 |
| *NPFE* | FEV_0.05_ | mL | 1.06 ± 0.06 | 0.81 ± 0.12 *** | 0.77 ± 0.13 *** | 0.94 ± 0.06 ** | 0.99 ± 0.06 |
|  | FEV_0.1_ | mL | 1.28 ± 0.07 | 0.98 ± 0.19 *** | 1.12 ± 0.20 * | 1.22 ± 0.08 | 1.21 ± 0.08 |
|  | FEV_0.2_ | mL | 1.37 ± 0.07 | 1.05 ± 0.21 *** | 1.46 ± 0.24 | 1.31 ± 0.09 | 1.29 ± 0.08 |
|  | FVC | mL | 1.40 ± 0.06 | 1.08 ± 0.22 ** | 1.82 ± 0.19 *** | 1.34 ± 0.09 | 1.33 ± 0.07 * |
|  | FEV_0.05_/FVC | % | 75.23 ± 2.26 | 76.41 ± 7.95 | 42.06 ± 4.21 *** | 70.24 ± 4.27 * | 74.68 ± 2.57 |
|  | FEV_0.1_/FVC | % | 91.48 ± 2.01 | 91.51 ± 3.03 | 61.20 ± 6.20 *** | 90.58 ± 3.07 | 90.81 ± 2.62 |
|  | FEV_0.2_/FVC | % | 97.47 ± 0.93 | 97.21 ± 1.05 | 80.05 ± 6.17 *** | 97.67 ± 0.91 | 96.98 ± 2.07 |
|  | FEF_0.05_ | mL/s | 8.27 ± 0.64 | 6.27 ± 2.50 * | 9.81 ± 2.22 | 9.65 ± 1.32 * | 7.92 ± 1.14 |
|  | FEF_0.1_ | mL/s | 2.12 ± 0.44 | 1.50 ± 0.86 | 5.25 ± 1.19 *** | 2.48 ± 0.62 | 2.16 ± 0.64 |
|  | FEF_0.2_ | mL/s | 0.28 ± 0.09 | 0.27 ± 0.11 | 2.26 ± 0.57 *** | 0.29 ± 0.13 | 0.30 ± 0.15 |
|  | PEF | mL/s | 42.21 ± 3.50 | 33.96 ± 3.63 *** | 24.83 ± 6.20 *** | 37.92 ± 2.80 * | 37.49 ± 4.97 |

PVr-P: ramp-style, pressure-driven PV loop, NPFE: negative pressure-driven forced expirations.

Data are represented as mean ± SD. *p < 0.05 and ***p < 0.001 compared to the naive control group. n = 7-8 per group.
