# Supplementary material for: Forced expiration measurements in mouse models of obstructive and restrictive lung diseases
Source: Respir Res. 2017 Jun 19;18:123. doi: 10.1186/s12931-017-0610-1 (PMC5477381; doi:10.1186/s12931-017-0610-1)
Supplement: Supplementary file 3 — Forced expiration-derived parameters. Negative pressure-driven forced expiration maneuvers were performed at baseline and following each methacholine aerosol challenge (0 –20 mg/mL). FEV0.1/FVC ratio is shown for LPS-ALI (a) and HDM-asthma (b). The mean (± SD) concentration-response curve of each group is represented together with the naive control group. n = 7 –8 per group. (PPTX 131 kb) [file 12931_2017_610_MOESM3_ESM.pptx]

## Slide 1
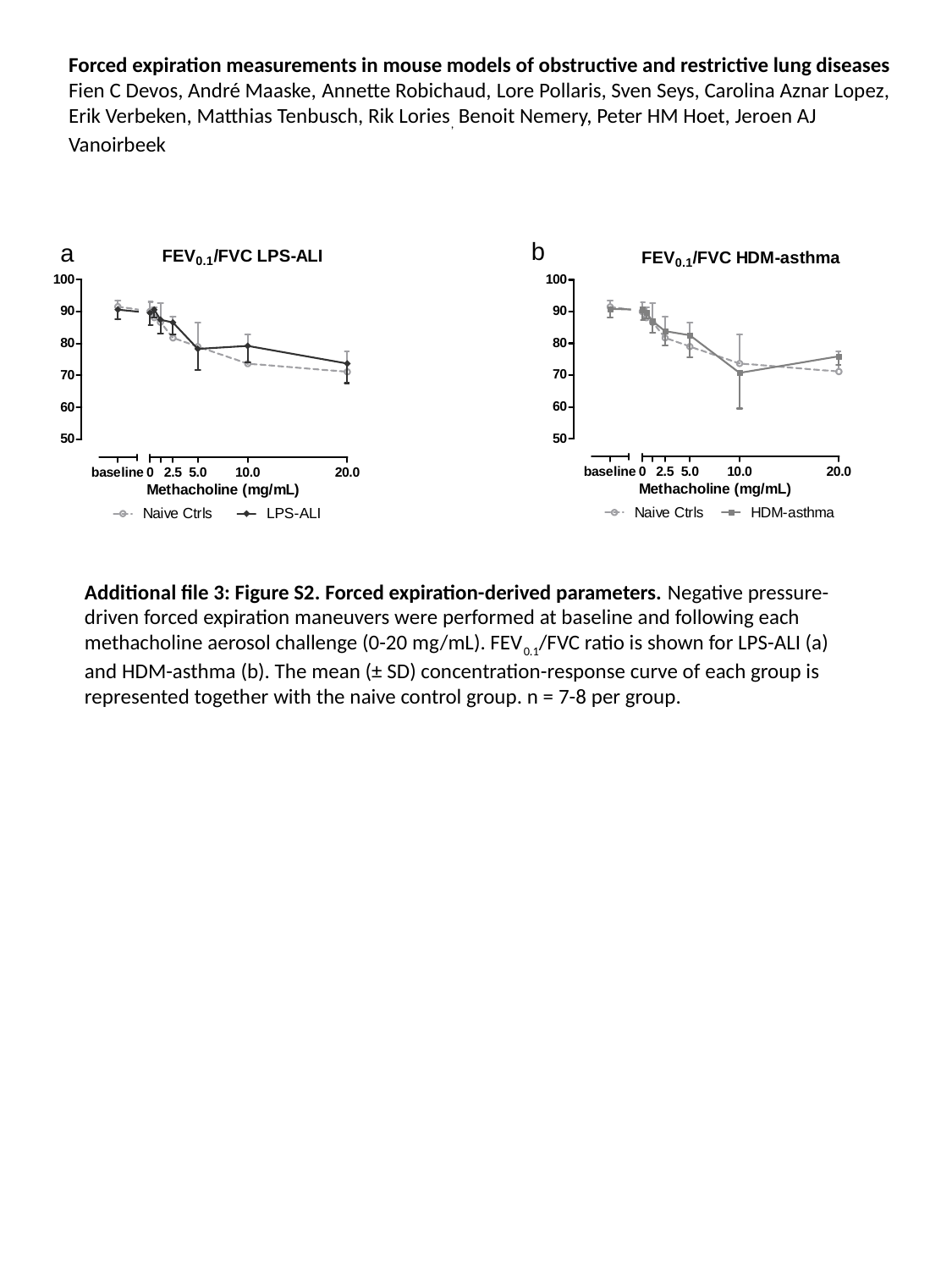

Forced expiration measurements in mouse models of obstructive and restrictive lung diseases
Fien C Devos, André Maaske, Annette Robichaud, Lore Pollaris, Sven Seys, Carolina Aznar Lopez, Erik Verbeken, Matthias Tenbusch, Rik Lories, Benoit Nemery, Peter HM Hoet, Jeroen AJ Vanoirbeek
b
a
Additional file 3: Figure S2. Forced expiration-derived parameters. Negative pressure-driven forced expiration maneuvers were performed at baseline and following each methacholine aerosol challenge (0-20 mg/mL). FEV0.1/FVC ratio is shown for LPS-ALI (a) and HDM-asthma (b). The mean (± SD) concentration-response curve of each group is represented together with the naive control group. n = 7-8 per group.
